# A Bioinformatics Pipeline for the Analysis and Target Prediction of RNA Effectors in Bidirectional Communication During Plant–Microbe Interactions

**DOI:** 10.3389/fpls.2018.01212

**Published:** 2018-08-20

**Authors:** Silvia Zanini, Ena Šečić, Lukas Jelonek, Karl-Heinz Kogel

**Affiliations:** ^1^Institute of Phytopathology, Centre for BioSystems, Land Use and Nutrition, Justus Liebig University Giessen, Giessen, Germany; ^2^Institute of Bioinformatics and Systems Biology, Justus Liebig University Giessen, Giessen, Germany

**Keywords:** small RNA, cross-kingdom RNAi, bidirectional communication, RNA targets, plant disease, virulence

## Abstract

Small RNA (sRNA) molecules are key factors in the communication between hosts and their interacting pathogens, where they function as effectors that can modulate both host defense and microbial virulence/pathogenicity through a mechanism termed cross-kingdom RNA interference (*ck*-RNAi). Consistent with this recent knowledge, sRNAs and their double-stranded RNA precursor have been adopted to control diseases in crop plants, demonstrating a straight forward application of the new findings to approach agricultural problems. Despite the great interest in natural *ck*-RNAi, it is astonishing to find just a few additional examples in the literature since the first report was published in 2013. One reason might be that the identification of sRNA effectors is hampered both by technical challenges and lack of routine bioinformatics application strategies. Here, we suggest a practical procedure to find, characterize, and validate sRNA effectors in plant–microbe interaction. The aim of this review is not to present and discuss all possible tools, but to give guidelines toward the best established software available for the analysis.

## Introduction

Natural cross-kingdom RNA interference (*ck*-RNAi) is an emerging field of research in plant–microbe interactions and plant pathology (Cai et al., 2018). The phenomenon includes small RNA (sRNA) (Borges and Martienssen, 2015) that are mutually transferred between interacting hosts and pathogens to eventually target and thus modulate respective host defense and pathogen virulence functions by RNAi (Weiberg et al., 2013; Zhang et al., 2016). RNAi is the process by which 21–24 nucleotide (nt) sRNAs are used by ARGONAUTE (AGO) proteins to guide an RNA-Induced Silencing Complex (RISC) toward a complementary messenger RNA (mRNA), resulting either in mRNA cleavage and degradation, or impairment of its transcription by acting as a physical block (Fire et al., 1998; Hamilton and Baulcombe, 1999).

Recent studies have shown that fungal pathogens can produce and deliver sRNAs to host plants in order to suppress their immunity and thus aid colonization (Weiberg et al., 2013; Wang B. et al., 2017). During infection, *Botrytis cinerea* transfers sRNAs to Arabidopsis and tomato cells, targeting host genes known to be involved in plant defense responses, including transcription factors and receptor-like kinases (Wang M. et al., 2017). This silencing is possible due to the fungus hijacking the RNAi machinery of the host, in particular AtAGO1. Consequently, the fertile hypomorphic *ago1-27* Arabidopsis mutant shows increased resistance to *Botrytis* infection. Interestingly, the *ago1-27* mutant is similarly less infected compared to wild type (wt) plants when infected with *Verticillium dahliae* (Vd), suggesting a role for RNAi also in this interaction.

Furthermore, the causal agent of stripe rust in wheat, *Puccinia striiformis* (*Ps*), delivers fungal microRNA (miRNA)-like RNAs into host cells to suppress the defense response by targeting and downregulating wheat *Pathogenesis-related 2* (*PR-2*) expression. Silencing of the sRNA precursor showed enhanced resistance to the virulent *Ps* isolate in wheat adult plants (Wang B. et al., 2017).

Preliminary results indicate the bidirectionality of this process in the interaction between Vd and cotton plants. In Vd samples recovered from infected cotton plants, 28 different sRNAs were predicted to originate not from Vd, but from the cotton plant, implying that host-derived sRNAs had been transferred into the pathogen during infection (Zhang et al., 2016). Despite the great interest in natural *ck*-RNAi and its agronomic application (Koch et al., 2013, 2016; Koch and Kogel, 2014; Wang et al., 2016; Niehl et al., 2018), it is astonishing to find just a few examples in the literature since the first report was published by Weiberg and colleagues in 2013 (Weiberg et al., 2013). One reason might be that the identification of sRNA effectors is hampered both by technical challenges and lack of routine bioinformatics application strategies. Here we suggest a practical procedure to find, characterize, and validate sRNA effectors in plant–microbe interactions. The aim of this review is not to present and discuss all possible tools, but to give guidelines toward the establishment of a suitable pipeline for the analysis.

## Strategies to Find, Characterize, and Validate sRNA Effectors in Plant–Microbe Interactions

### Sample Preparation and sRNA Sequencing

In all cases, identification and confirmation of cross-kingdom sRNAs (*ck*-sRNAs) starts with the preparation of suitable biological samples. When planning which and how many samples to sequence, control samples of uninfected plants and, when possible, axenic cultures of the microbe should be included in order to verify the infection-related upregulation/induction of the candidate *ck*-sRNAs in bidirectional fashion. The preparation also requires to fix the number of replicates and of reads per sample (typically three biological replicates with 5–10 million reads each, Conesa et al., 2016).

While sequencing can be carried out using a variety of machines, the focus of this review is on sequencing with Illumina technology. With TruSeq Small RNA Library Preparation by Illumina it is possible to create indexed libraries of sRNAs both from total RNA and from pre-size-selected fractions, depending on the need to preserve mRNA for further sequencing or not. Libraries can then be pooled and sequenced on various Illumina systems, including MiSeq and NextSeq, depending on the output range and total reads per run required. Subsequent analysis of this data is the critical point toward discovery of candidate *ck*-sRNAs. There are numerous detailed papers comparing the performances of the programs mentioned in this review, which are recommended for reading once a preliminary pipeline is chosen, as the fine tuning of the software settings is specific for each project/organism analyzed (Dai et al., 2011a; Srivastava et al., 2014; Li et al., 2015; Conesa et al., 2016).

Although known *ck*-sRNAs are between 21 and 24 nt long (Weiberg et al., 2013; Wang M. et al., 2017; Wang B. et al., 2017), the range for the size selection can be increased to 18–35 nt for detection of all other known regulatory sRNAs. Sequencing depth, number of replicates, and type of libraries are all experiment-specific and highly variable depending on the aim of the study and the resources available. For example, for adequate statistical power in the data analysis, a minimum of three biological replicates is required (Love et al., 2014) and, while for mRNA it is usually recommended to explore the option of longer read length or paired-end (PE) sequencing, single-end (SE) short reads are perfectly suitable for sRNAseq. Regardless of the specific datasets, some measures have to be taken to ensure the removal of unwanted fragments that would overweigh the sequences of interest. In particular, size selection prior to sRNA sequencing is required to avoid sequencing longer fragments that would not be the focus of the study.

### Determination of Candidate *ck*-sRNA

Acquisition of the raw reads is the first step of the bioinformatics analysis and is immediately followed by quality check. FastQC (Andrews, 2010) is the most frequently used program for this task, as it is recommended by Illumina for the analysis of Illumina NGS data and it is compatible also with PacBio and 454 datasets. Alternatively, programs such as NGS-QC can also be used for the analysis of data obtained from several sequencing platforms (Dai et al., 2010). With these the overall quality of the sequencing can be assessed, in particular the sequence quality, GC content, N content, and overrepresented sequences. While there are no universal cutoffs for some of these tests, as the values vary based on the organisms and the sequencing setups, similar results should be obtained throughout the same kind of datasets both for “failed” k-mer and duplication tests.

**FIGURE 1:**
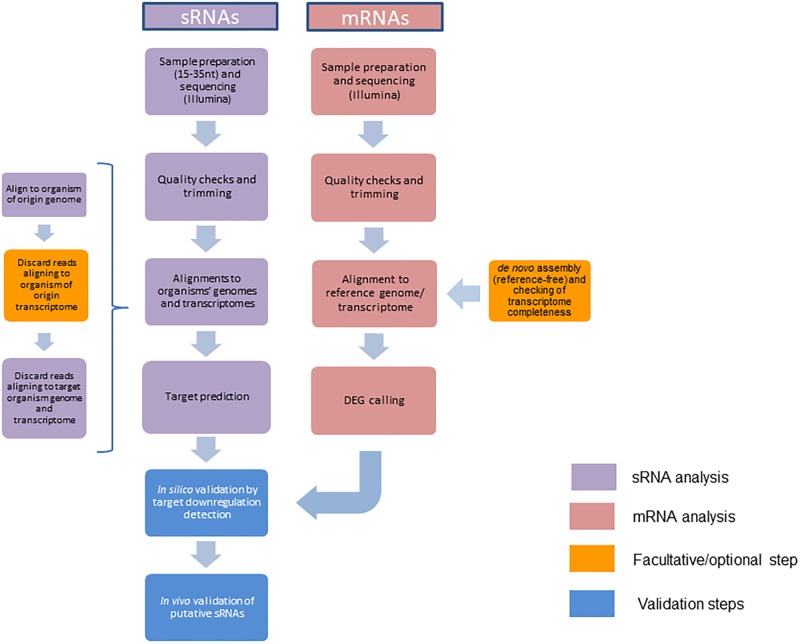
Bioinformatics pipeline for the detection and validation of cross-kingdom (*ck*)-RNA effectors in interactions of plant hosts with microbial pathogens.

When analyzing sRNA datasets, a test that will most likely fail is the adapter content test: given that the fragments sequenced (usually 18–35 nt) are often shorter than the read length (36 bp), the machine is bound to read into the adapter. There are number of software programs designed to do the necessary trimming, such as Cutadapt or FASTX-Toolkit (Martin, 2011). At this point of the analysis, low quality reads/bases should be removed, as well as adapters and PCR artifacts. The workflow proposed, summarized in **Figure 1**, can be used to identify the sRNAs originating from either the host or the microbe by assigning the “sRNA source” organism and the “sRNA target” organism at the beginning of the analysis. Afterward, the same pipeline can be applied with the roles inverted to obtain information about both sides of the bidirectional cross-kingdom communication.

Trimmed reads can now be mapped with short read un-gapped mappers such as Bowtie (Langmead, 2010) and SOAP (Li et al., 2008) to reference genomes and transcriptomes; by this way one gains information on the origin of the potential *ck*-sRNA and its localization in the respective genome. Given that it is crucial to find the sRNAs from the source organism that target the interacting organism, this alignment step includes the removal of reads that align to both organisms. In particular, sRNAs are kept only if (i) they align 100% of the full read length to the source organism’s genome (bowtie settings: -v0 –al), and (ii) have at least two mismatches to the target organism’s genome or transcriptome (bowtie settings: -v1 –un). As an additional step, the removal of sequences aligning 100% to the source organism’s transcriptome can be done in order to select exclusively sRNAs originating from non-coding regions (bowtie settings: -v0 –un), removing short sequences derived from mRNA degradation. After the alignments, sRNAs can be additionally filtered based on their presence in the sample of the pure source organism as they are expected to be either upregulated in the sample from the interacting organisms compared to the control (pure organism), or present exclusively in that sample (Weiberg et al., 2013).

### *ck*-sRNA Target Detection and Evaluation

The remaining sequences can be further analyzed for target prediction. There are various software platforms available for small interfering RNA (siRNA) and microRNA (miRNA) detection, originally tailored for mammal sRNAs. While these can be customized for plant and microbe studies, two well established tools are designed specifically for plants: psRNATarget and TAPIR (Bonnet et al., 2010; Dai and Zhao, 2011b). Both are comparable regarding sRNA identification rates at their default settings and are widely used in plant miRNA analysis and discovery research, making them the best options for this analysis. While TAPIR offers a standalone and an online version, psRNATarget is only available online, making it less convenient for automatized workflows. On the other hand, psRNATarget provides more options for customizing settings and parameters of the prediction, making it more adaptable to different organisms and systems. Both programs work by aligning sRNA sequences to the target transcriptome and assigning penalties for mismatches, gaps, and G:U pairs, in particular in the seed region (between positions 2 and 12 of the sRNA for TAPIR and 2–13 for psRNATarget), which is critical for target recognition. The resulting score is between 0 and 5, and can be decreased from the default value to further reduce the risk of false positives. Additionally, TAPIR separately scores the free energy ratio, represented by the free energy of the predicted sRNA:target duplex divided by the free energy of the corresponding duplex having a perfect complementarity (Bonnet et al., 2010). In this case, the minimal value cutoff suggested is 0.7 (range between 0 and 1). The default output of both programs is a table containing all scores of the sRNA:mRNA duplexes, the alignment itself and, if available, a description of the target mRNA.

### Analysis of Target Transcript Expression

The first validation step of candidate *ck*-sRNAs is the confirmation of target gene downregulation in the colonized tissue as a result of the cleavage of the corresponding mRNA by the RISC enzymatic complex. Plant and microbial mRNA levels can be checked by mRNA sequencing analysis from the same biological samples the sRNA was obtained from. Since the library preparation for sRNA libraries is based on size separation and excision of a specific nt length interval, the longer RNA fraction from the same samples can be used to prepare mRNA libraries. Read length and sequencing depth selected can vary depending on the experimental design and resources available, but the mRNA sequences are primarily obtained from the large RNA fraction after polyA affinity selection, as more than 90% of total RNA is comprised of ribosomal RNA (rRNA) (Conesa et al., 2016). In bacterial samples or in samples with low RNA integrity number (RIN) values, where polyA selection would not be effective, rRNA depletion can be done instead. The bioinformatic pipeline described in the following paragraph will serve as a primary *in silico* validation step toward confirmation of candidate *ck*-sRNA activity in the target organism.

Since there is a multitude of RNAseq tools available, the experimental design and the availability of published sequence data are the main factors in deciding on a pipeline (Conesa et al., 2016). Quality check and trimming of sequencing artifacts are necessary steps at the beginning of the analysis, following the similar principle as in sRNA analysis, namely the use of FastQC (Andrews, 2010) and cutadapt (Martin, 2011). If reference sequences for the organisms involved are available, the mapping of RNAseq reads can be done as a straight forward strategy (see below). Depending on available -omics data for the organisms in question, mapping of reads can be done to the reference genome or transcriptome. Without available reference sequences, *de novo* assembly (reference-free) of the transcriptome can be computed from all RNAseq datasets, usually with a De Bruijn graph-based assembler like Trinity (Grabherr et al., 2011), SOAPdenovo-*Trans* (Xie et al., 2014), Oases (Schulz et al., 2012), or *Trans*-AbySS (Robertson et al., 2010). Functional annotation and ortholog search can then be performed with common platforms such as BLAST (Altschul et al., 1990) and ENSEMBL (Zerbino et al., 2018) or, specifically developed but harder to install, transcriptome annotation tools like Trinotate (github^1^) and FunctionAnnotator (Chen et al., 2017). Transcriptome completeness can be checked with Busco (Simão et al., 2015). These tools can also be used in case of unsatisfactory annotation of available reference genome or transcriptome.

Spliced alignment to the reference genome is done by mappers that take into account the introns in the genome. TopHat/TopHat2 (Trapnell et al., 2009; Kim et al., 2013) are gapped mappers developed to detect novel splice-sites. They were superseded by a new mapper called HISAT2 (Kim et al., 2015) that is more accurate and much more efficient. Another option is Spliced Transcripts Alignment to a Reference (STAR), which also allows for fast and precise mapping with known and novel splice-sites (Dobin et al., 2013). Correction for exon sizes specific to the respective plant and microbe organism in question are needed, since typically the programs use default settings for the human genome. Un-gapped mappers, such as Bowtie (Langmead, 2010), can be used to map against a reference transcriptome if no novel transcript discovery is needed. However, since the goal of the analysis is to discover a high number of transcripts, including those with a low level of expression, and since organisms in question often are not sufficiently annotated, the gapped mapping on a genome followed by quality control is the recommended strategy. The quality of the mapping can be checked by programs such as Picard (Picard tools^2^, github), RseQC (Wang et al., 2012), and Qualimap (García-Alcalde et al., 2012). Percentage of mapped reads, multi-mapping reads (mapping to the several identical regions), and the uniformity of read coverage are relevant parameters to assess sequencing quality at this point (Conesa et al., 2016).

The first step for differential gene expression (DGE) is determining transcript abundances using program packages like Cufflinks (Trapnell et al., 2010) or htseq-count (Anders et al., 2015). DGE analysis can be done by a variety of programs, including DeSeq (Anders and Huber, 2010), Deseq2 (Love et al., 2014), edgeR (Robinson et al., 2010), and voom (Law et al., 2014). Low replicate numbers of transcripts and outliers among the replicates can complicate the DGE analysis. Thus, a powerful analytical method proves crucial to determine when the fold change in transcripts between the control and treated sample is different. The programs differ in statistical distributions they use for analysis of data and how they treat the variability among the replications, but a comparison study claims DeSeq2 and edgeR have an advantage when it comes to smaller number of replicates (below 12) (Schurch et al., 2016).

The results of this target prediction and analysis pipeline can be visualized at several levels and by a variety of programs, some of which focus on sRNA-mRNA duplex conformation structure and others on a broader representation of cross-kingdom effects between genomes. ReadXplorer (Hilker et al., 2014) and Integrative Genomics Viewer (IGV) (Thorvaldsdóttir et al., 2013) are used for different types of presentation of sequencing data, and in this case specifically for visualization of mapped reads on the reference genome. miRPlant (An et al., 2014), a tool for prediction of miRNAs from NGS data, provides the visual presentation of the predicted miRNA in the precursor hairpin structure and with the indication of where the mature fragment is. CummerBund (Goff et al., 2013) is commonly used after the Cufflinks package for visualization of differentially expressed genes in different types of plots. The software package Circos (Krzywinski et al., 2009) is a good choice for circular visualization of entire genomes, transcriptomes, sRNA candidates, and the range of silencing downregulation effects.

### Further Validation of *ck*-RNAs

Having obtained information on differentially expressed genes in the treated sample, the ones which are significantly downregulated and predicted as targets of candidate *ck*-sRNAs are investigated further. Alternative to mRNA sequencing, validation of *ck*-sRNA candidates requires confirmation of downregulation of their putative target by qRT-PCR.

Additional *in vivo* validation of the interaction of *ck*-sRNA candidates with their targets can be based on genetic analysis, e.g., mutational knockdown (KO) strategies of target genes and/or precursor loci of candidate *ck*-sRNAs. Finally, we suggest that the following additional analyzes are required to unequivocally claim a *ck*-RNA-mediated target interaction in *ck*-RNAi: (i) verification of sRNA-target interaction in transient expression systems such as leaves of *Nicotiana benthamiana*; (ii) testing respective AGO and DCL mutants for a loss of *ck*-RNA function in RNAi-mediated target downregulation; and (iii) detection of direct association of *ck*-RNAs or their target mRNA with the respective microbial or plant AGO1 protein by immuno-purification techniques (Jain et al., 2011; Riley et al., 2012; Carbonell, 2017).

## Conclusion

RNAi-based bidirectional communication between interacting organisms, also called *ck*-RNAi, has been detected in a few natural plant – microbe systems, but the implications of a novel effector class of sRNA are significant. So far, there is no evidence that the activity of such RNA effectors would be restricted to certain microbial life styles as they were identified in plants interacting with both biotrophic and necrotrophic microbes. The recent finding that mammals also generate RNA effectors to combat parasites suggests that the phenomenon is widespread and prevalent in different types of host–parasite interaction. Besides the exciting discovering of a novel chemical communication strategy, the knowledge on *ck*-RNAs opens new avenues in sustainable and environmentally safe plant protection as sRNAs and their cellular precursors dsRNAs are natural molecules with an anti-microbial activity. However, the detection and validation of RNA-based communication and *ck*-RNAi still relies on the available data about model species and a narrow range of investigated systems. The present review tries to give a practical outline of a pipeline for *ck*-RNA detection focused on plant – microbe systems. A bioinformatics pipeline used to strengthen and accelerate the experimental approaches is of paramount importance for confirmation of sRNA communication between plants and microbes in a multitude of relevant systems.

## Author Contributions

SZ, K-HK, EŠ, and LJ wrote the text. SZ and EŠ designed the figure.

## Conflict of Interest Statement

The authors declare that the research was conducted in the absence of any commercial or financial relationships that could be construed as a potential conflict of interest.

## Footnotes

1 http://trinotate.github.io/

2 http://broadinstitute.github.io/picard
